# Lymphocytic Choriomeningitis Virus Bearing GPC Mutations for Enhanced Tumor Tropism and Strong Anti-Tumor Activity

**DOI:** 10.3390/v18090998

**Published:** 2026-09-11

**Authors:** Sarah-Kim Friedrich-Becker, Michael Bergerhausen, Arshia Berry, Hilal Bhat, Fanghui Li, Rosa Schmitz, Lisa Holnsteiner, Dethardt Müller, Haifeng Xu, Philipp A. Lang, Karl Sebastian Lang, Jörg Vollmer

**Affiliations:** 1 Abalos Therapeutics GmbH, 40225 Düsseldorf, Germany; bergerhausen@abalos-tx.com (M.B.); arshia.berry@precisionformedicine.com (A.B.); mueller@abalos-tx.com (D.M.); vollmer@abalos-tx.com (J.V.); 2 Institute of Immunology, University Hospital of Essen, 45147 Essen, Germany; bhathilal673@gmail.com (H.B.); 15927580042@163.com (F.L.); lisa.holnsteiner@uk-essen.de (L.H.); karlsebastian.lang@uk-essen.de (K.S.L.); 3 National Center for Radiation Research in Oncology, Faculty of Medicine and University Hospital Carl Gustav Carus, TUD Dresden University of Technology, 01309 Dresden, Germany; 4 Department of Molecular Medicine II, Medical Faculty, Heinrich Heine University, 40225 Düsseldorf, Germany; xuh@hhu.de (H.X.); langp@uni-duesseldorf.de (P.A.L.)

**Keywords:** adaptive immunity, cancer, cold tumors, hot tumors, innate immunity, LCMV, tumor microenvironment, viroimmunotherapy

## Abstract

The development of effective viroimmunotherapies for cancer relies on the ability of replicating viruses to infect the entire tumor tissue and induce comprehensive inflammation within the tumor microenvironment (TME). In this study, we investigate the impact of mutations in the glycoprotein (GP) of lymphocytic choriomeningitis virus (LCMV) on tumor cell infectivity, replication, and the immune response. Specifically, we focus on two key positions in GP1 (181 and 185) that significantly enhance tumor cell infectivity and improve CD8^+^ T cell function in the GP-mutated LCMV strain LCMV P52. Our findings demonstrate that LCMV strain LCMV P52 replicates preferentially in vivo in tumors, exhibits improved tumor penetration, attracts functional CD8^+^ T cells more effectively to the tumor, and reprograms the tumor toward a more immunogenic microenvironment to allow for effective checkpoint inhibitor combination.

## 1. Introduction

Cancer, a multifaceted and heterogeneous disease, continues to challenge clinicians and researchers worldwide. Despite significant advancements in targeted therapies and immunotherapies, there remains an unmet need for effective treatments, especially in aggressive and refractory malignancies.

Solid tumors differ widely in immune infiltration and therapy responsiveness: “hot” immunogenic tumors, enriched with activated T cells, respond relatively well to immunotherapy, whereas “cold” non-immunogenic tumors with immunosuppressive microenvironments remain largely resistant [1,2]. Immunovirotherapy can convert cold tumors into hot ones by inducing immunogenic tumor cell death and recruiting immune effector cells [3,4,5]. Yet many commonly used oncolytic viral platforms (e.g., adenovirus, HSV, and vaccinia virus) have limited capacity for systemic spread due to pre-existing immunity, eradication by complement activation or other anti-viral immune mechanisms. This limits durable antigen expression, and non-replicating and attenuated vectors especially tend to generate only transient short-lived immune activation [6,7,8]. In contrast, replication-competent lymphocytic choriomeningitis viruses (LCMVs) can better resist anti-viral immunity, persist longer in tumor tissues, sustain antigen release, and continuously engage both innate and adaptive immune mechanisms.

LCMV is a non-cytopathic bi-segmented RNA virus of the family *Mammarenaviridae*. Its genome is organized into two RNA segments, the large (L) and small (S) segments [9]. The L segment encodes the RNA-dependent RNA polymerase and the RING finger protein (Z). The S segment encodes the two viral glycoproteins, GP1 and GP2, and the nucleoprotein [10,11]. The trimeric GP proteins of LCMV build the virus envelope and facilitate binding to surface receptors of host cells [12,13]. Mutations in the glycoproteins can highly influence the ability to infect cells and can alter the tropism of the virus [14,15,16].

The LCMV wildtype strain WE has been shown to have a strong anti-tumoral effect against solid cancers [17]. Due to its natural tropism towards tumors and antigen-presenting cells, it preferentially replicates in tumor tissue and activates the innate immune system by inducing type I interferons (IFN-Is) [17,18]. Despite having direct apoptotic and proliferation-inhibiting potential, type I IFNs play a crucial role in the remodeling of the tumor microenvironment (TME). The resulting type I IFNs reprogram the tumor microenvironment, promoting efficient activation and expansion of virus- and tumor-specific CD8^+^ T cells that mediate anti-tumor effects and play major roles in anticancer immune responses [19,20,21,22,23].

Previously, mutations I181M and R185W, among others, in the GP protein of wildtype strain LCMV WE were shown to be critical for virus infection and the induction of an effective anti-virus and anti-tumor immune response [16]. The resulting virus variant named LCMV P52 exhibited enhanced tumor cell tropism and improved CD8^+^ T cell function [24]. In the present study, we investigated the safe intravenous administration and the anti-tumoral properties of LCMV P52 in more detail across multiple murine tumor models differing in baseline immunogenicity, including immune-inflamed (“hot”) tumors and immune-excluded (“cold”) tumor models. We comprehensively analyzed viral replication as well as short-term and long-term anti-tumor effects, including remodeling of the tumor microenvironment. In addition, we explored combinatorial strategies employing LCMV P52 with PD-1 blockade or adoptive tumor-specific T cell transfer to assess whether viral inflammation synergizes with checkpoint or cellular immunotherapy.

## 2. Materials and Methods

### 2.1. Cell Lines

MC38-Ova and TRAMP-C2 were purchased from ATCC (Manassas, VA, USA) and cultured in Dulbecco’s Modified Eagle Medium (DMEM) with 10% fetal bovine serum (FBS). CT26 and 4T1 cells were purchased from ATCC and cultured in RPMI-1640 medium with 10% fetal bovine serum (FBS). B16-Ova cell line was kindly gifted from P. Knolle (Technical University of Munich, Munich, Germany) and cultured in DMEM with 10% fetal bovine serum in the presence of 1 µg/mL Puromycin (Invivogen, San Diego, CA, USA).

### 2.2. Virus Stock Generation

For virus stock production, BHK-21 cells were cultured in a T175 flask to 60–70% confluence in DMEM with 5% FBS. Medium was removed from adherent cells and replaced by 5 mL DMEM with 2% FBS containing 2 × 10^4^ FFU of the desired virus. After 20 min of incubation at 37 °C, additional 25 mL DMEM with 2% FBS was added, and cells were incubated for 48 h. Thereafter, supernatant was transferred into a 50 mL tube and centrifuged for 20 min at 3220× *g* at 4 °C to remove cell debris. The clarified supernatant was then aliquoted and stored at −80 °C. Before the stock was used, virus titer was determined by chromogenic focus-forming assay and diluted to desired concentrations and verified by focus-forming assay.

### 2.3. Chromogenic Focus-Forming Assay (CFFA)

LCMV titers were determined by a chromogenic focus-forming assay on MC57-G cells. For titer determinations in organs, organs were smashed in DMEM with 2% FBS and 1% Penicillin/Streptomycin (PAN-Biotech, Aidenbach, Germany) using a tissue lyzer. Viral titers of supernatants of LCMV-infected tumor cells were determined directly. All the samples were serially diluted (1:3) over 12 dilutions in 96-well plates and transferred to 1 × 10^5^ MC57-G cells seeded in 24-well plates. After incubation for 4 h at 37 °C, cells were overlaid with a 1:1 mixture of 2% methylcellulose and 2× IMDM. Plates were incubated for 48 h at 37 °C and afterwards fixed with 4% Formalin. After permeabilization with 1% Triton X-100 and blocking with 10% FBS, staining was performed with primary rabbit anti-LCMV NP antibody (clone VL-4, in-house) and a secondary anti-rabbit-IgG-HRP antibody (Jackson ImmunoResearch, West Grove, PA, USA). LCMV foci were visualized by chromogenic color reaction (50 mM Na_2_HPO_4_, 25 mM citric acid, 0.03% H_2_O_2_, 0.4 mg/mL O-phenylenediamine dihydrochloride; Sigma-Aldrich, St. Louis, MO, USA).

### 2.4. Fluorescent Immunohistochemistry Staining

OCT (optimal cutting temperature) medium embedded samples were cut into 7 µm sections with a cryomicrotome (Leica, Nussloch, Germany) and transferred to glass slides. Samples were fixed with Acetone for 10 min at room temperature. Sections were surrounded by a hydrophobic barrier pen. Non-specific binding sites were blocked with PBS with 2% FBS for 15 min at room temperature. Fluorescently labeled primary antibodies (anti-CD8 (clone 53-6.7, BioLegend, San Diego, CA, USA)) or non-labeled primary antibodies (anti-LCMV NP from rat (clone VL4, in-house or BioXcell, Lebanon, NH, USA)) were diluted in PBS with 2% FBS and incubated with the samples for 30–60 min at room temperature. After incubation, samples were carefully washed with PBS with 2% FBS. Fluorescently labeled secondary antibodies (anti-rat-IgG (Jackson) and anti-rabbit-IgG (BioLegend)) were diluted in PBS with 2% FBS and incubated with samples for 30–60 min at room temperature. DAPI was used to visualize nuclei and either added to the primary or secondary antibody cocktail. Samples were washed, mounted with mounting medium (Dako, Glostrup, Denmark) and covered with coverslips. Sample analysis was performed with a fluorescence microscope (Keyence, Osaka, Japan).

### 2.5. Mice

C57BL/6J and OT-I mice were bred in-house. BALB/c were purchased from Animal Resource Centre (Canning Vale, WA, Australia). All the animals were healthy and had not undergone any previous experimental procedures or treatments prior to study inclusion. All the animal experiments were performed according to the German laws for animal protection and were authorized by the Landesamt für Natur-, Umwelt- und Klima NRW (LANUK) Nordrhein-Westfalen and/or the South Australian Health and Medical Research Institute Animal Ethics Committee. All the mice were housed in individually ventilated cages, checked daily, and sacrificed with corresponding termination criteria or due to experiment settings. The termination criteria were body weight, general condition, spontaneous behavior, clinical findings, and tumor size. For all the experiments 6–8-week-old mice were used. Mice were allowed to acclimatize for at least 6–8 days before the start of the experiment. Sample size was selected in accordance with the 3R principles and limited to the minimum number of animals required to obtain scientifically meaningful data. Given the exploratory nature of the analysis and the anticipated effect size, three animals per group were considered sufficient. Treatments and measurements were conducted in a balanced order where possible.

### 2.6. Measurement of Clinical Chemistry Parameters

The serum levels of alanine aminotransferase (ALT), aspartate aminotransferase (AST), alkaline phosphatase (ALP), creatine kinase (CK), lactate dehydrogenase (LDH), urea, triglycerides, and calcium were determined by the Central Laboratory of the University Hospital Essen. Prior to analysis, 30 µL of each serum sample was diluted 1:10 in PBS.

### 2.7. Subcutaneous Tumor Engraftment, Randomization and Blinding

The described cell lines were harvested from cell culture flasks and counted. Until injection, the cells were stored on ice to ensure high viability of injected cells. Then, 100 µL cell suspension was injected subcutaneously into an anesthetized mouse. After the tumor became palpable the mice were treated as indicated. The subcutaneous tumors were measured three times per week with a Castroviejo caliper. Animals were allocated to treatment groups either by randomization using a matched-pair distribution method based on tumor size or manual group assignment to ensure comparable mean baseline tumor volumes across groups. No blinding was performed. Investigators were aware of group allocation during group assignment, conduct of the experiments, outcome assessment, and data analysis.

### 2.8. Metastasis Model

5 × 10^5^ B16-Ova cells were intravenously injected into C57BL/6 mice on day 7, respectively. Mice were treated with 5 × 10^6^ OT-1 splenocytes i.v. on day 4 and infected with 1 × 10^5^ FFU of LCMV P52 or left untreated. Mice were sacrificed on day 9 after infection and lungs were removed to analyze metastasis formation.

### 2.9. Tetramer Staining of Blood Samples

Mice were bled into tubes with anticoagulant. Samples were incubated with fluorescently labeled Ova-tetramer for 15 min at 37 °C and afterwards stained for CD3 (clone 17A2, BioLegend) and CD8 (clone 53-6.7, BioLegend) in FACS Buffer for 30 min at 4 °C. Next, FACS Lysing Solution (BD Bioscience, San Jose, CA, USA) was added and incubated for 7 min at room temperature. Samples were washed with FACS buffer and analyzed by flow cytometry (Cytoflex, Beckmann Coulter, Brea, CA, USA).

### 2.10. Flow Cytometry of Tumor Tissue

Fresh tumor tissues were harvested, chopped into small pieces and digested for 30 min at 37 °C in RPMI containing 50 U/mL DNase I and 1 mg/mL collagenase A. Afterwards, red blood cells were lyzed using BD Pharm Lyse Buffer (BD Pharmingen, San Diego, CA, USA). After staining of cell surface markers with antibodies against CD3 (clone 17A2, BioLegend), CD4 (clone GK1.5, BioLegend), CD8 (clone 53-6.7, BioLegend), KLRG1 (clone 2F1/KLRG1, BioLegend), F4/80 (clone T45-2342, BD Biosciences), CD49b (clone DX5, BioLegend), CD25 (clone PC61, BioLegend), CD11b (clone M1/70, BD Biosciences), PD-1 (clone 29F.1A12, eBiosciences, San Diego, CA, USA) or CD11c (clone N418, BioLegend), samples were fixed and permeabilized using Transcription Factor Buffer Set (BD Biosciences) according to the manufacturer’s recommendations. Finally, intranuclear FoxP3 (clone R16-715, BD Biosciences) was stained, and samples were analyzed by flow cytometry.

### 2.11. Intracellular Cytokine Staining

After sacrifice of mice, spleens or tumor-draining lymph nodes were harvested and smashed through a 70 µm cell strainer. Single-cell suspensions were resuspended in DMEM 2% FBS and 1% Penicillin/Streptomycin and divided on 96-well U-bottom plates. DMEM containing the indicated peptide at 1 µg/mL or DMEM without any addition as a control was added. Samples were incubated for 1 h at 37 °C. Afterwards 10 µg/mL Brefeldin A (Sigma-Aldrich) was added to each well, and cells were incubated overnight. Cells were centrifuged and then incubated with anti-CD8 antibody (clone 53-6.7, BioLegend) for 30 min at 4 °C. Cells were washed with FACS Buffer and fixed with 2% Formalin for 10 min at room temperature. After washing, cells were incubated with fluorescently labeled antibodies against IFNγ (clone XMG1.2, BioLegend) and TNFα (clone MP6-XT22, BioLegend) in FACS buffer containing 0.1% saponin (Sigma-Aldrich) for permeabilization for 45 min at 4 °C. After a final wash, samples were analyzed using a flow cytometer (Cytoflex, Beckmann Coulter).

### 2.12. RT-qPCR

Tumors of mice were harvested and homogenized in TRIzol reagent. RNA isolation was performed using the DirectZol Mini Kit (Zymo Research, Irvine, CA, USA) according to the manufacturer’s protocol. RT-qPCR was performed using a 1-step RT-qPCR System (Promega, Madison, WI, USA) and gene-specific primers (Ifna4 QT01774353, Ifnb1 QT00249662, Gzmb QT00114590, and Gapdh QT01658692). CT values were normalized to Gapdh expression in untreated controls.

### 2.13. Generation of LCMV GP 3D Model

The prediction of the LCMV glycoprotein surface was calculated with PyMol (Python version 3.14) by aligning the artificial dimeric crystal structure of LCMV GP published by [25] (PDB 5ine) with the trimeric GP structure of the closely related Lassa virus (PDB 5vk2, [26]).

### 2.14. Statistical Analysis

Data presentation and statistical analysis were performed with GraphPad Prism (version 10). Ordinary one-way ANOVA was used to compare a single outcome across more than two independent groups, whereas two-way ANOVA was used when the outcome was assessed across two experimental factors; Welch’s *t*-test was used for comparisons between two independent groups, as indicated. No formal assessment of statistical assumptions was performed. A *p*-value < 0.05 was regarded as statistically significant. No predefined inclusion or exclusion criteria were established for the animals. Data points were excluded from the analysis only if a manual recording or data entry error was identified.

## 3. Results

### 3.1. Serial Passaging of LCMV WE on Lung Tumor Cells Results in GP Mutations

**Figure 1:**
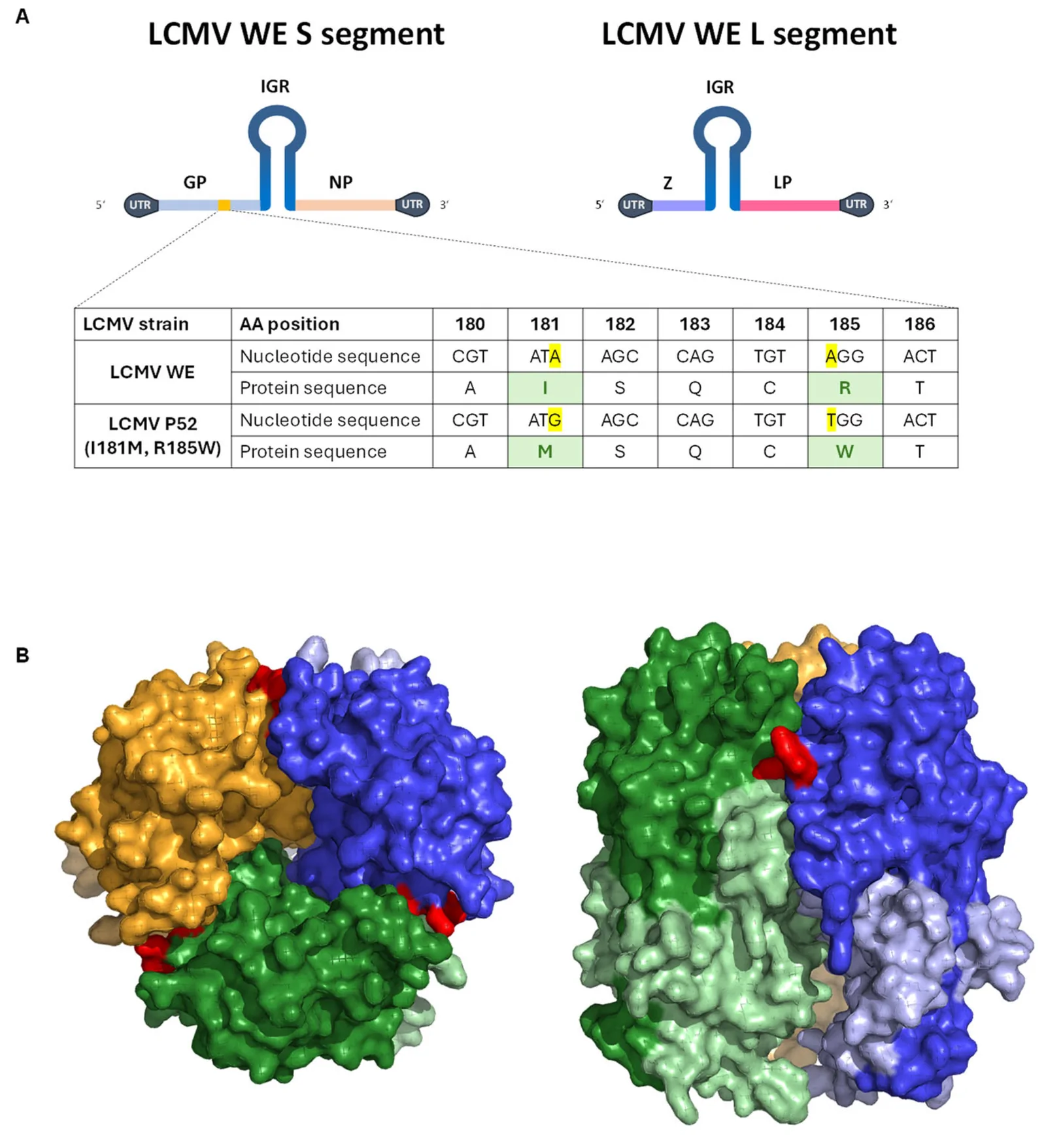
Genomic and structural organization of the LCMV glycoprotein and location of tumor-adaptive glycoprotein mutations. (**A**) The LCMV genome consists of an S segment encoding glycoprotein (GP) and nucleoprotein (NP) and an L segment encoding the polymerase (LP) and Z protein. Serial passaging of parental LCMV WE on human lung tumor cells resulted in the emergence of mutations I181M and R185W within the GP gene, first detected after 52 passages (LCMV P52). The nucleotide substitutions and resulting amino acid changes are shown below. Yellow highlights indicate nucleotide changes; green highlights indicate amino acid changes. (**B**) 3D structure of the LCMV GP trimer from top (**left**) and side (**right**) views. The positions of I181M and R185W are highlighted in red.

The virus used in this study, hereafter referred to as LCMV P52, was generated through serial passaging of the parental LCMV WE strain on human lung tumor cells. During this adaptation process, two amino acid substitutions in the viral glycoprotein (GP), I181M and R185W, emerged and were first detected after 52 passages, leading to the designation LCMV P52 (Figure 1). Previous studies have demonstrated that the introduction of GP mutations can enhance viral entry into both healthy and tumor cells [27]. In contrast, the mutations present in the LCMV P52 variant have been shown to enhance the infectivity of tumor cells while reducing infection of plasmacytoid dendritic cells (pDCs), leading to enhanced CD8^+^ T cell function [24].

### 3.2. LCMV P52 Virotherapy Inflames Cold Tumor Microenvironments

**Figure 2:**
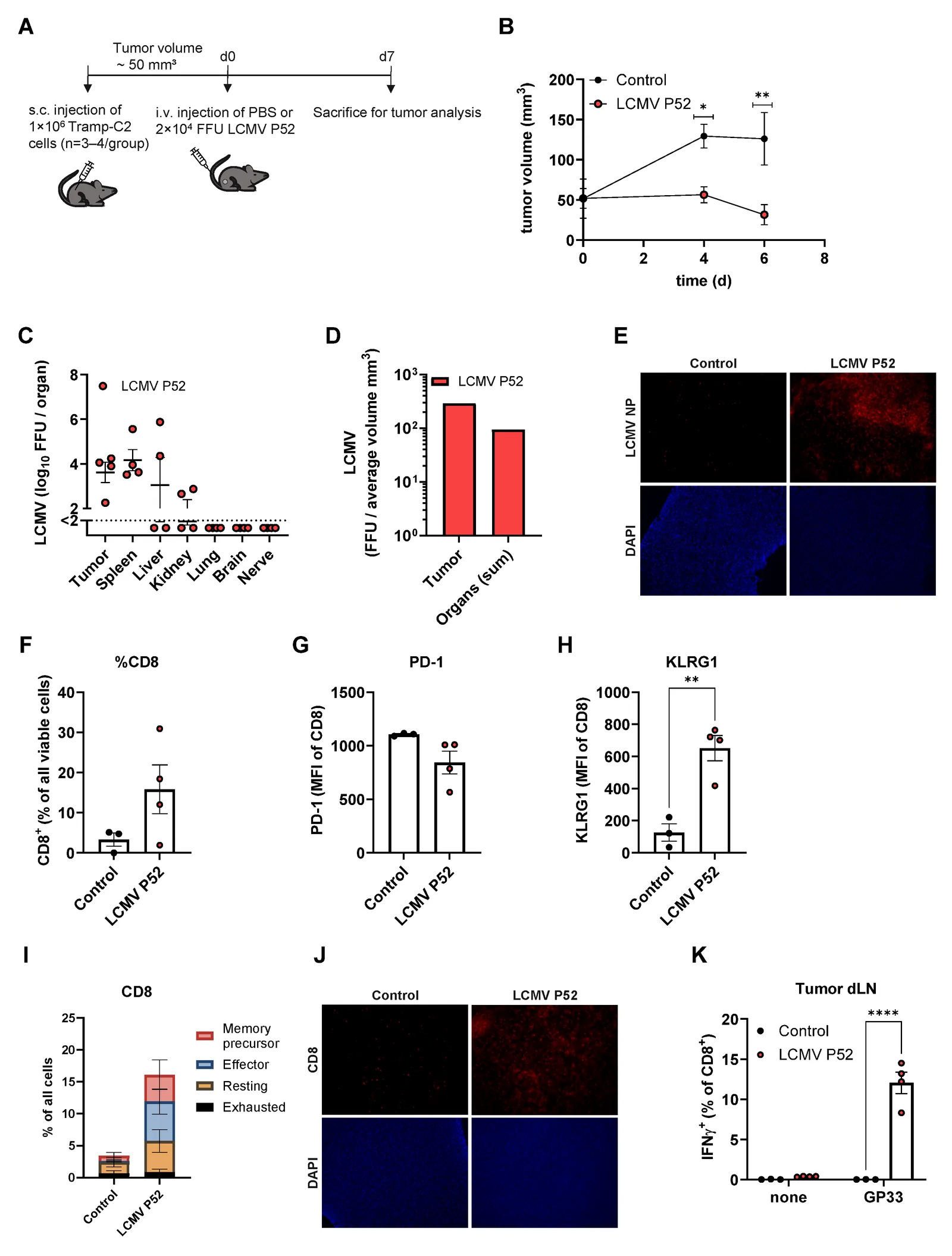
LCMV P52 virotherapy ignites CD8^+^ immunity and suppresses growth in a prototypical cold tumor model. (**A**–**K**) Male C57BL/6 mice were subcutaneously injected with 1 × 10^6^ Tramp-C2 tumor cells. Once tumors reached ~50 mm^3^, mice were treated intravenously on day 0 with PBS as a control treatment (black circles, *n* = 3) or infected intravenously with 2 × 10^4^ FFU of LCMV P52 (red circles, *n* = 4). (**B**) Tumor volume (mm^3^) was measured twice a week until day 6 after infection. (**C**) Seven days after treatment, organs and tumor tissue of LCMV P52 (*n* = 4) were analyzed for infectious virus titers by CFFA. (**D**) Ratio of infectious LCMV P52 virus particles to sum of average organ volume of organs analyzed in (**C**) or tumor volume at endpoint as shown in (**B**). (**E**) Tumor sections (7 µm) of PBS-treated controls (*n* = 3) or LCMV P52-treated mice (*n* = 4) were stained by fluorescent immunohistochemistry for LCMV NP (red) and DAPI (blue) and visualized by fluorescence microscopy. One representative image per condition is shown. (**F**–**I**) Flow cytometry analysis of tumor tissue of PBS-treated controls (*n* = 3) or LCMV P52-treated mice (*n* = 4) showing the percentage of CD8^+^ T cells and the mean fluorescence intensity (MFI) of PD-1 and KLRG1 on CD8^+^ T cells and the distribution among exhausted (KLRG1^+^PD-1^+^), resting (KLRG1^−^PD-1^−^), effector (KLRG1^+^PD-1^−^) and memory precursor (KLRG1^−^PD-1^+^) subtypes. (**J**) Tumor sections (7 µm) were stained by fluorescent immunohistochemistry staining for CD8 (red) and DAPI (blue). (**K**) CD8^+^ T cells of PBS-treated controls (*n* = 3) or LCMV P52-treated mice (*n* = 4) from tumor-draining lymph nodes were restimulated ex vivo in the absence of peptide or in the presence of the LCMV GP33 peptide. Interferon-γ (IFNγ) production was measured by intracellular cytokine staining followed by flow cytometric analysis. One representative picture per condition is displayed. Mean ± SEM. (**B**,**C**,**F**–**H**,**K**) Dotted line represents assay lower limit of detection. * *p* ≤ 0.05, ** *p* ≤ 0.01, **** *p* ≤ 0.0001; two-way ANOVA (**B**,**K**); Welch’s *t* test (**F**–**H**). Non-significant differences are not indicated.

As enhanced viral entry of the LCMV P52 variant has been shown to promote robust T cell responses [24], we hypothesized that preferential tumor infection by LCMV P52 may facilitate immune activation within otherwise immunologically cold tumors. To test this, we first investigated the short-term anti-tumoral effects of LCMV P52 in the Tramp-C2 prostate tumor model (Figure 2A). The short observation period was selected to assess early immunological and virological responses, such as CD8^+^ T cell infiltration and viral replication, which are transient and predominantly detectable within the first week following infection before being controlled by the host immune system [28,29].

LCMV P52 displayed strong anti-tumoral efficacy compared to the control treatment, with a tendency towards tumor regression early upon infection (Figure 2B). We further analyzed the presence of infectious virus in tumors and different organs, which are known target organs for LCMV replication [30,31,32,33] (Figure 2C). LCMV P52 was readily detectable in tumor tissue as well as in the spleen, liver and kidney on day 7 after infection. In addition, we calculated the infectious virus titers of LCMV P52 in tumors and organs in relation to the average volume of these organs (measured for tumors at the final day of the experiment or obtained from the literature for the other organs [34]) (Figure 2D). Our data underlines that LCMV P52 exhibits more pronounced virus replication in tumors compared to the total replicating virus particles detected in all the other healthy target organs. Visualization of LCMV P52 virus particles in tumor by immune fluorescence staining further demonstrated the distribution of virus across the entire tumor tissue (Figure 2E). To assess whether replication of LCMV P52 contributes to inflammation of the cold tumor microenvironment, we analyzed CD8^+^ T cell infiltration and activation within the tumor tissue. Our data show an increase in CD8^+^ T cells in Tramp-C2 tumors after LCMV P52-treatment (Figure 2F). These T cells displayed a modestly reduced exhaustion profile, reflected by lower PD-1 expression (Figure 2G), together with a significant increase in T cell activation, marked by elevated KLRG1 expression (Figure 2H). To further investigate the activation state of infiltrating CD8^+^ T cells, they were classified into four distinct subsets based on their expression of KLRG1 and PD-1. While exhausted T cells (KLRG1^+^PD-1^+^) remained almost unchanged under LCMV P52 treatment, the infiltrating T cells exhibited effector (KLRG1^+^PD-1^−^) or memory precursor phenotypes (KLRG1^−^PD-1^+^). Additionally, the expansion of yet resting cells (KLRG1^−^PD-1^−^) may indicate de novo priming of CD8^+^ T cells in the tumor microenvironment (Figure 2I). The immunofluorescence analysis revealed that CD8^+^ T cells were distributed throughout the entire tumor tissue (Figure 2J). We further examined virus antigen-specific responses to LCMV P52 by restimulating T cells from tumor-draining lymph nodes. Restimulation induced robust interferon-γ (IFNγ) production in virus-specific CD8^+^ T cells (Figure 2K). In summary, LCMV P52 accumulates and replicates preferentially in tumors, driving CD8^+^ influx and activation, expanding antigen-specific responses in tumor-draining lymph nodes, and suppressing tumor growth in a cold tumor model.

### 3.3. LCMV P52 Drives Potent CD8^+^ T Cell Infiltration into Hot Tumors

**Figure 3:**
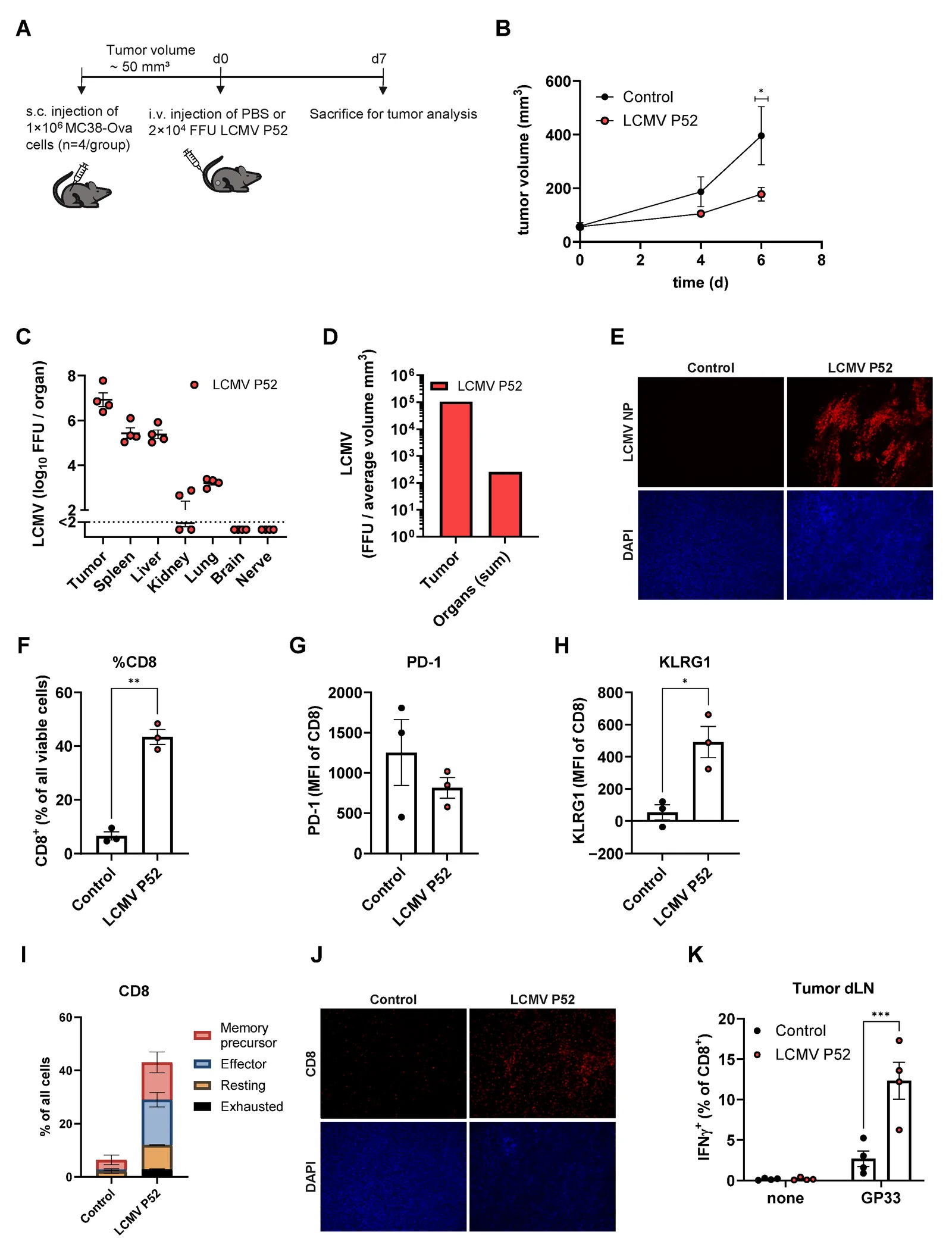
LCMV P52 amplifies pre-existing immunity in hot tumors, driving robust CD8^+^ T cell recruitment and tumor control in a prototypical hot tumor model. (**A**–**K**) Female C57BL/6 mice were subcutaneously injected with 1 × 10^6^ MC38-Ova tumor cells. When tumors reached ~50 mm^3^, mice were treated intravenously on day 0 with PBS as control (black circles, *n* = 4) or infected intravenously with 2 × 10^4^ FFU of LCMV P52 (red circles, *n* = 4). (**B**) Tumor volume (mm^3^) was monitored daily and measured twice a week until day 7 post-infection. (**C**) Seven days after treatment, organs and tumor tissue of LCMV P52-treated mice (*n* = 4) were collected and analyzed for infectious virus titers by CFFA. (**D**) Tumor sections (7 µm) of PBS-treated controls (*n* = 4) or LCMV P52-treated mice (*n* = 4) were stained by fluorescent immunohistochemistry for LCMV NP (red) and DAPI (blue) and visualized by fluorescence microscopy. One representative image per condition is shown. (**E**) Ratio of infectious LCMV P52 virus particles to the sum of average organ volumes (as quantified in panel (**C**)) or to tumor volume at endpoint as shown in panel (**B**). (**F**–**H**) Flow cytometry analysis of PBS-treated controls (*n* = 3 ^#^) or LCMV P52-treated mice (*n* = 4) of tumor tissue showing the percentage of CD8^+^ T cells and mean fluorescence intensity (MFI) of PD-1 and KLRG1 on CD8^+^ T cells. (**I**) Distribution of exhausted (KLRG1^+^PD-1^+^), resting (KLRG1^−^PD-1^−^), effector (KLRG1^+^PD-1^−^) and memory precursor (KLRG1^−^PD-1^+^) CD8^+^ T cell subtypes. (**J**) Tumor sections (7 µm) of PBS-treated controls (*n* = 4) or LCMV P52-treated mice (*n* = 4) were stained for CD8 (red) and DAPI (blue) by fluorescent immunohistochemistry. One representative image per condition is displayed. (**K**) CD8^+^ T cells from tumor-draining lymph nodes of PBS-treated controls (*n* = 4) or LCMV P52-treated mice (*n* = 4) were restimulated ex vivo either without peptide or with the LCMV GP33 peptide. Interferon-γ production was assessed by intracellular cytokine staining followed by flow cytometry. Mean ± SEM. (**B**,**C**,**F**–**I**,**K**) * *p* ≤ 0.05, ** *p* ≤ 0.01, *** *p* ≤ 0.001; two-way ANOVA with Šidák correction for multiple comparisons (**B**,**K**). Unpaired two-tailed Welch’s *t* test (**F**–**H**). Non-significant differences are not indicated. ^#^ Of the four control and treatment samples, only three could be analyzed due to insufficient material available from one control sample.

We next sought to determine whether the early anti-tumoral and immunomodulatory effects observed in the cold Tramp-C2 model could be extended to tumors with a pre-existing inflamed (“hot”) tumor microenvironment (TME). As in the previous experiment, the short observation period was chosen to capture early events, including viral replication and immune cell infiltration, before viral control by the host immune response. Therefore, we treated mice bearing the hot MC38-Ova tumor either with PBS as a control or 2 × 10^4^ FFU of LCMV P52 (Figure 3A). The treatment with LCMV P52 resulted in significant tumor growth inhibition by day 6 post-infection (Figure 3B). We further investigated the distribution of replicating LCMV P52 particles in tumor and LCMV healthy target organs on day 7 p.i. (Figure 3C). Virus replication was detected in tumor tissue, as well as in spleen, liver, kidney and lung. In accordance with data from the Tramp-C2 model, the highest virus titers per mm^3^ of organ were detectable in tumor tissue (Figure 3D). We further visualized the distribution of LCMV P52 particles in tumors. LCMV P52 was distributed throughout the entire tumor tissue, with certain areas representing active replication (Figure 3E). To determine whether intratumoral replication of LCMV P52 also promotes T cell infiltration in already inflamed tumor tissues, we analyzed CD8^+^ T cells by flow cytometry. To our surprise, the CD8^+^ T cell infiltration into hot MC38-Ova tumors was also significantly increased compared to the control treatment (Figure 3F). This increase was even more pronounced than in the Tramp-C2 non-inflamed tumor model. Consistent with our earlier findings, the infiltrating CD8^+^ T cells displayed reduced PD-1 expression (Figure 3G) and a significantly elevated activation state, marked by increased KLRG1 expression (Figure 3H). Again, mainly effector, memory precursor and resting T cells could be found, although the number of exhausted cells increased slightly, which might be a result of ongoing inflammation in the tumor tissue. In accordance with the flow cytometry data, immunofluorescence staining of tumor tissue further underlined the increased influx of CD8^+^ T cells (Figure 3J). We next assessed antigen-specific responses to LCMV P52 by restimulating T cells isolated from the tumor-draining lymph nodes (Figure 3K). Upon GP33 restimulation, virus-specific CD8^+^ T cells produced robust amounts of IFNγ. Together, these findings demonstrate that LCMV P52 maintains its tumor-selective replication while restraining tumor growth, reshaping the immune microenvironment, and amplifying systemic antigen-specific T cell responses in both cold and hot tumor models, thereby establishing a multifaceted anti-tumor mechanism.

### 3.4. LCMV P52 Infection Results in Longer-Term Anti-Tumor Effects

**Figure 4:**
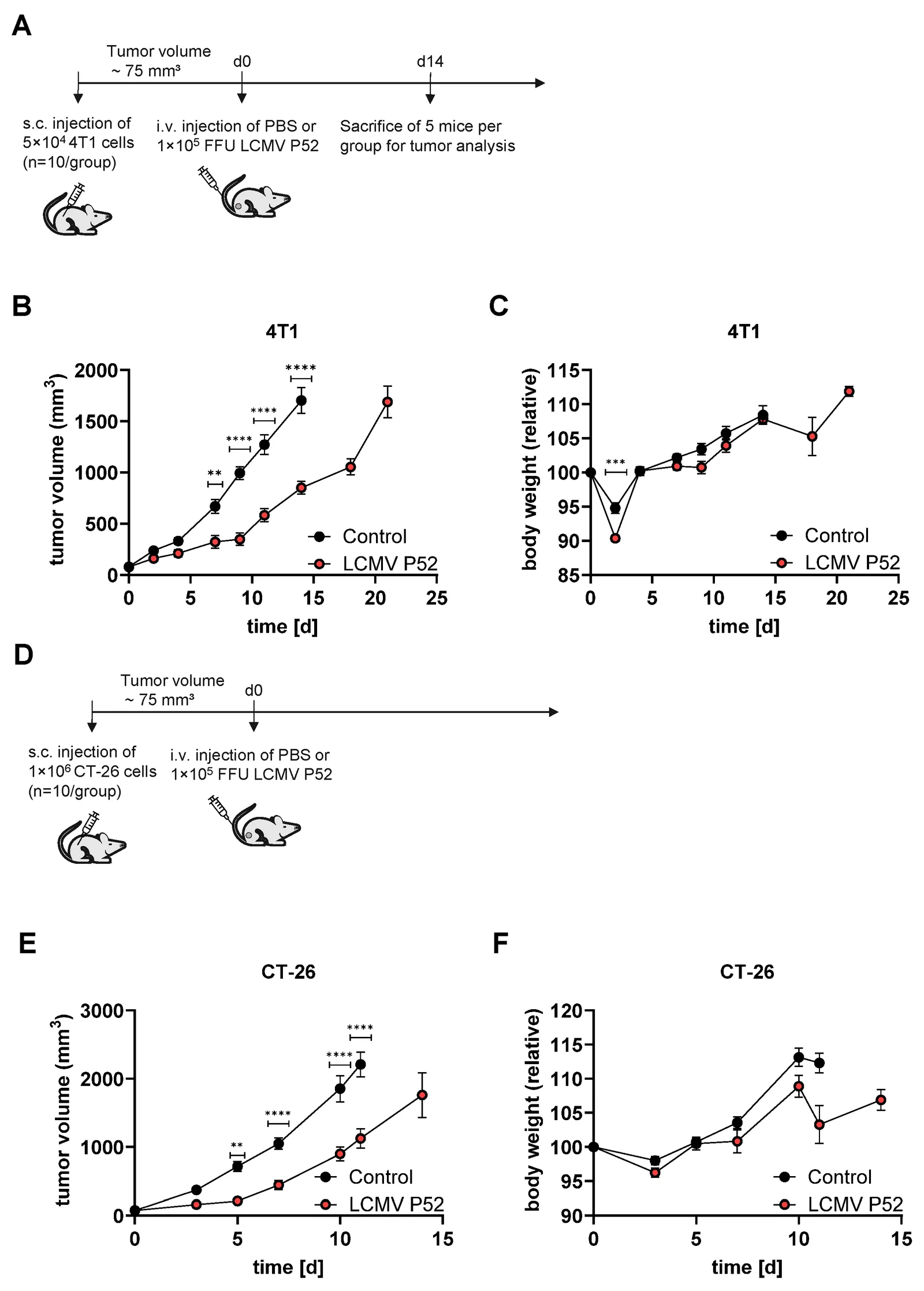
LCMV P52 achieves long-term tumor control in immune-desert 4T1 and immunogenic CT26 tumors. (**A**–**C**) Female BALB/c mice were subcutaneously injected with 5 × 10^4^ 4T1 tumor cells. Upon a tumor volume of ~75 mm^3^, mice were i.v. treated with PBS as control treatment (black circles, *n* = 10) or i.v. infected with 2 × 10^4^ FFU of LCMV P52 (red circles, *n* = 10) on day 0. Tumor volume ((**B**), mm^3^) and body weight (**C**) were measured every two to four days until day 14 (PBS, *n* = 10) or day 21 (LCMV P52, *n* = 10) after infection. (**D**–**F**) Female BALB/c mice were subcutaneously implanted with 1 × 10^6^ CT-26 tumor cells. Once tumors reached ~75 mm^3^, mice were treated intravenously with PBS (black circles, *n* = 10) or 2 × 10^4^ FFU of LCMV P52 (red circles, *n* = 10) on day 0. Tumor volume (**E**) and body weight (**F**) were monitored every two to four days until day 11 for PBS-treated mice (*n* = 10) and until day 14 for LCMV P52-treated mice (*n* = 10). Mean ± SEM. (**B**,**C**,**E**,**F**) ** *p* ≤ 0.01, *** *p* ≤ 0.001, **** *p* ≤ 0.0001; two-way ANOVA with Šidák correction for multiple comparisons until day 14 (4T1) or day 11 (CT-26) (**B**,**E**). Non-significant differences are not indicated.

We further analyzed the longer-term anti-tumoral effects and potential adverse effects (by determining body weight) of LCMV P52 in the immunologically cold 4T1 and hot CT-26 tumor models (Figure 4A,D). Even though LCMV P52 exhibited stronger short-term anti-tumoral effects in the cold Tramp-C2 model compared to the hot MC38-Ova model (tumor growth inhibition (TGI) by LCMV P52 on the final day of the experiment: Tramp-C2 = 75% and MC38-Ova = 55%), LCMV P52 showed comparable tumor growth reduction in the cold 4T1 (Figure 4B) and hot CT-26 models (Figure 4E). Tumor volume was reduced by LCMV P52 treatment to approximately 50% TGI in both models (day 14: 4T1; day 11: CT-26). Interestingly, mild decreases in body weight were observed for both the control group and the LCMV P52 treatment group in the 4T1 model on day 2 after the start of treatment, which might be explained by stress in the mice due to the randomization process (Figure 4C). The relatively higher body weight loss observed in the LCMV P52-treated group compared to the control group may, at least partially, be induced by type I interferons, which are well documented to contribute to body-weight loss during LCMV infection [35,36]. As the observed weight loss was transient and resolved without intervention, LCMV P52 remains consistent with an overall favorable safety profile. LCMV P52 also displayed a beneficial safety profile without striking body weight loss upon treatment in the hot CT-26 tumor model (Figure 4F). Overall, LCMV P52 therapy achieves sustained tumor control in both 4T1 and CT26 tumors without signs of substantial systemic adverse effects.

### 3.5. LCMV P52 Induces Substantial Changes to More Immunogenic Tumor Microenvironments

**Figure 5:**
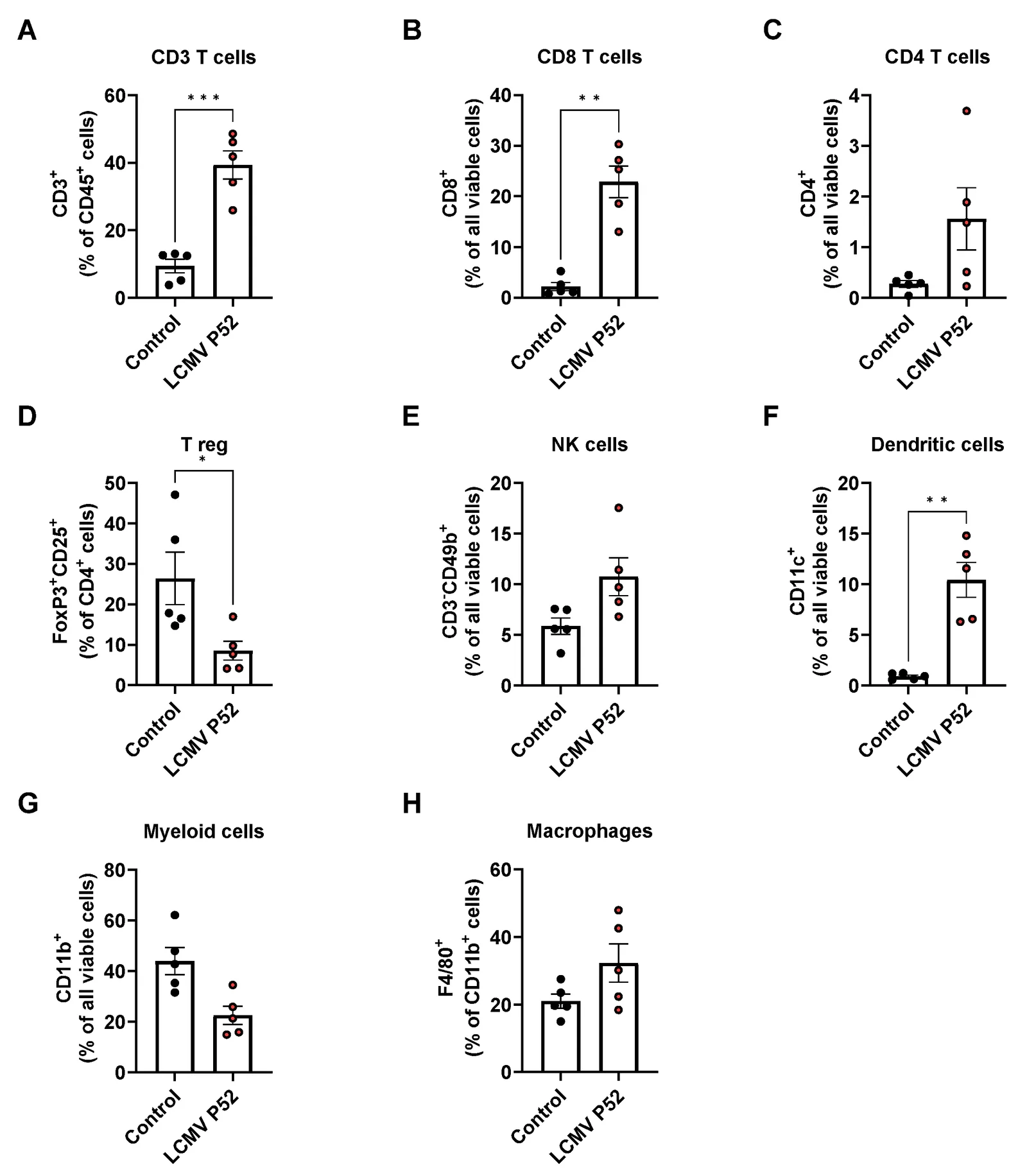
LCMV P52 reprograms the immune-deserted 4T1 tumor microenvironment toward T cell dominance. (**A**–**H**) Female BALB/c mice were injected subcutaneously with 5 × 10^4^ 4T1 cells and treated intravenously with PBS (*n* = 5) or 2 × 10^4^ FFU of LCMV P52 (*n* = 5). 14 days after treatment mice were sacrificed and tumor tissue was analyzed by flow cytometry to determine the percentages of CD3^+^, CD4^+^, CD8^+^ T cell, regulatory T cell (Treg), NK cell, dendritic cell, myeloid cell, and macrophage populations. Mean ± SEM. (**A**–**H**) * *p* ≤ 0.05, ** *p* ≤ 0.01, *** *p* ≤ 0.001; unpaired two-tailed Welch’s *t* test (**A**–**H**). Non-significant differences are not indicated.

Our data underline a pronounced anti-tumoral effect in the cold 4T1 model during the long-term study. We therefore aimed to evaluate whether treatment with LCMV P52 results in sustained remodeling of the TME (Figure 5). The flow cytometry analysis of 4T1 tumors two weeks after infection showed highly increased CD3^+^ T cell levels (Figure 5A). The investigation of the T cell subpopulations revealed a significantly higher increase in cytotoxic CD8^+^ T cells (Figure 5B) compared to CD4^+^ T cells (Figure 5C), which were still mildly increased compared to control conditions. Even though CD4^+^ T cells tended to be increased, a strong reduction in regulatory CD4^+^ T cells was observed in response to LCMV P52 treatment (Figure 5D). We further analyzed other non-T cell immune cell populations in the TME. Increases in NK cells and macrophages, as well as a strong increase in dendritic cells, were observed (Figure 5E,F,H), whereas myeloid cells were decreased in LCMV P52-treated animals (Figure 5G).

We also investigated changes in the TME in the immunologically hot CT-26 tumor model. Even though solid tumor growth inhibition by LCMV P52 treatment was observed, TME remodeling was not as striking as in the cold tumor model (Supplementary Figure S1). A slight increase in CD3^+^ T cells was observed, with the effect being more pronounced in CD8^+^ T cells than in CD4^+^ T cells (Supplementary Figure S1A–C). No substantial changes were detected for regulatory T cells, NK cells, or myeloid cell populations (Supplementary Figure S1D,E,G). Consistent with the findings from the cold 4T1 tumor model, dendritic cells showed a modestly increased population (Supplementary Figure S1F). In conclusion, LCMV P52 drives strong anti-tumor efficacy in the cold 4T1 and hot CT26 tumor models. Especially in the cold 4T1 model, this is paralleled by profound immunological reprogramming of the TME, including a significant increase in intratumoral T cell populations.

### 3.6. LCMV P52 Fights Tumor Metastases and Enhances Anti-Tumor Activity of Tumor-Specific T Cells

**Figure 6:**
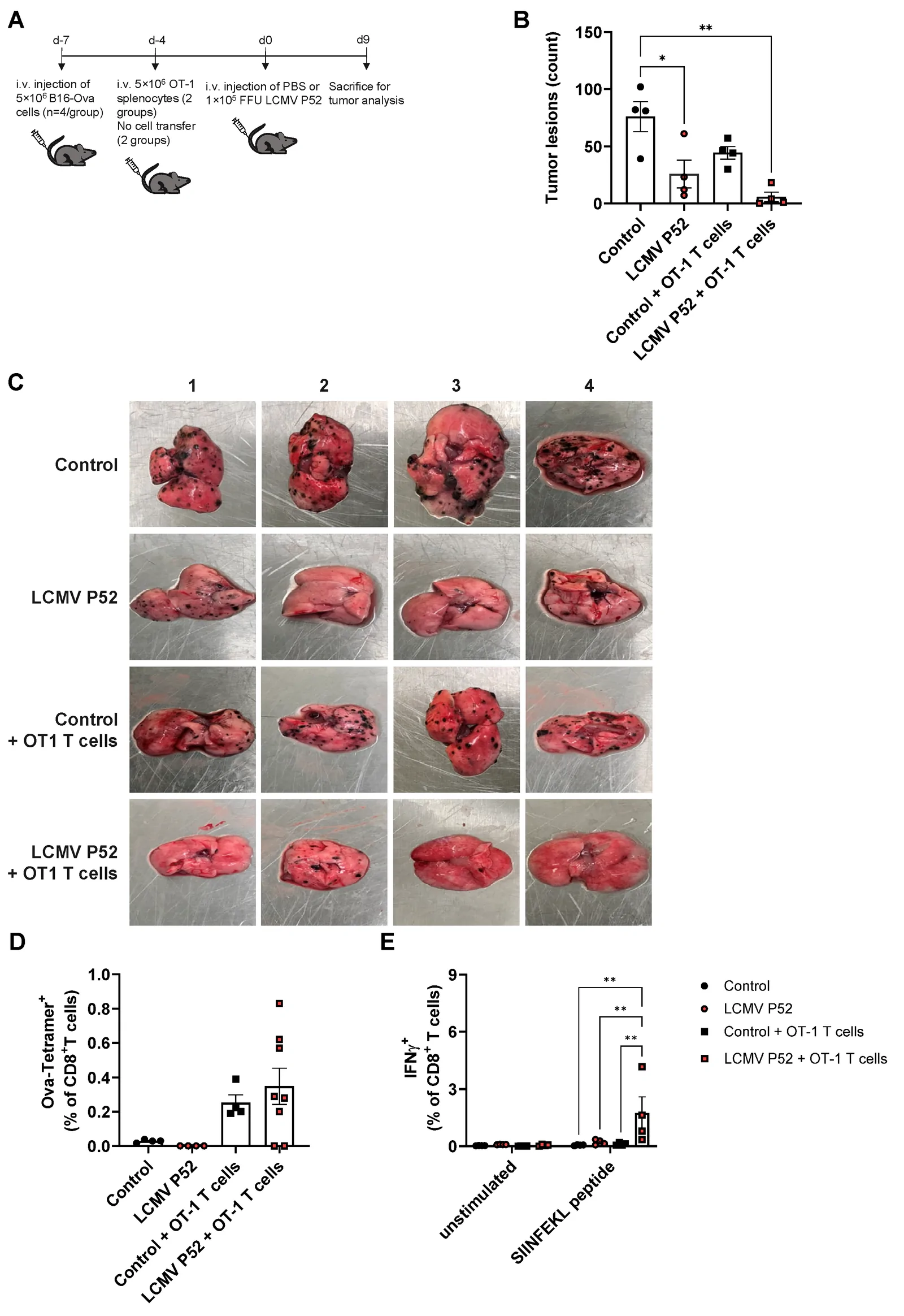
LCMV P52 virotherapy converts a non-permissive lung metastasis site into a T cell-responsive niche. (**A**–**E**) Female or male C57BL/6 mice were injected i.v. with B16-Ova tumor cells on day 7. On day 4, 5 × 10^6^ OT-1 splenocytes were transferred to two groups. On day 0, control mice (*n* = 4, upper panel (**C**), black circles (**D**,**E**)) and control mice with transferred OT-1 cells (*n* = 4, third panel (**C**), black squares (**D**,**E**)) were left untreated. 1 × 10^5^ FFU of LCMV P52 was i.v. injected into mice without transferred OT-1 cells (*n* = 4, second panel (**C**), red circles (**D**,**E**)) or with transferred OT-1 cells (*n* = 8, fourth panel (**D**), red squares (**D**,**E**)) on day 0. (**B**) Quantification of visible pulmonary tumor nodules on day 9 post-infection in mice treated as described above. (**C**) Representative images of lung metastases from mice described above on day 9 after infection. The black nodules visible on the lungs represent tumor lesions. (**D**) Percentage of Ova-tetramer-specific CD8^+^ T cells in blood of control mice (*n* = 4), LCMV P52-treated mice (*n* = 4), control mice + OT-1 T cells (*n* = 4) or LCMV P52-treated mice + OT-1 T cells (*n* = 8) on day 3 after infection. (**E**) Flow cytometry analysis of intracellular IFNγ production of spleen-derived CD8^+^ T cells either not stimulated with peptide or stimulated with SIINFEKL peptide (OVA-specific) from control mice (*n* = 4), LCMV P52-treated mice (*n* = 4), control mice + OT-1 T cells (*n* = 4) or LCMV P52-treated mice + OT-1 T cells (*n* = 4 ^#^). Mean ± SEM (**D**,**E**). Statistical analysis was performed with ordinary one-way ANOVA followed by Tukey’s multiple-comparisons test (**B**,**D**) or two-way ANOVA with Šidák correction for multiple comparisons (**E**). * *p* ≤ 0.05, ** *p* ≤ 0.01; values without statistical significance are not highlighted. ^#^ Due to sample processing constraints, spleen samples from four mice were selected for analysis.

Given the pronounced anti-tumor efficacy of LCMV P52 in subcutaneous tumor settings, we next explored its capacity to target metastatic lesions. For this purpose, we systemically introduced B16-Ova cells into C57BL/6 mice to assess the therapeutic potential of LCMV P52 in a model of disseminated tumor growth. To gain deeper insight into the effect of how LCMV P52 shapes tumor-specific CD8^+^ T cell responses, metastatic burden was compared in mice with or without adoptive transfer of Ova-specific OT-1 CD8^+^ T cells (Figure 6A). OT-1 T cell transfer and LCMV P52 infection affected the formation of metastatic lung B16-Ova lesions upon a single administration (Figure 6A,B). However, combining OT-1 T cell transfer with LCMV P52 treatment resulted in stronger effects on metastasis formation. This result suggests increased activity or presence of tumor-specific T cells in tumor metastases or tumor tissues. Indeed, tumor-specific Ova-tetramer^+^ T cells displayed slightly higher numbers in the blood of B16-Ova-bearing animals upon LCMV P52 infection (Figure 6D) and showed a markedly higher frequency of IFNγ-secreting tumor-specific CD8^+^ T cells (Figure 6E). In conclusion, our data point to accelerated tumor T cell penetration and increased adaptive anti-tumor immune mechanisms contributing to the LCMV P52 anti-tumor activity.

### 3.7. LCMV LCMV P52 Effectively Combines with Checkpoint Inhibitor Treatment

**Figure 7:**
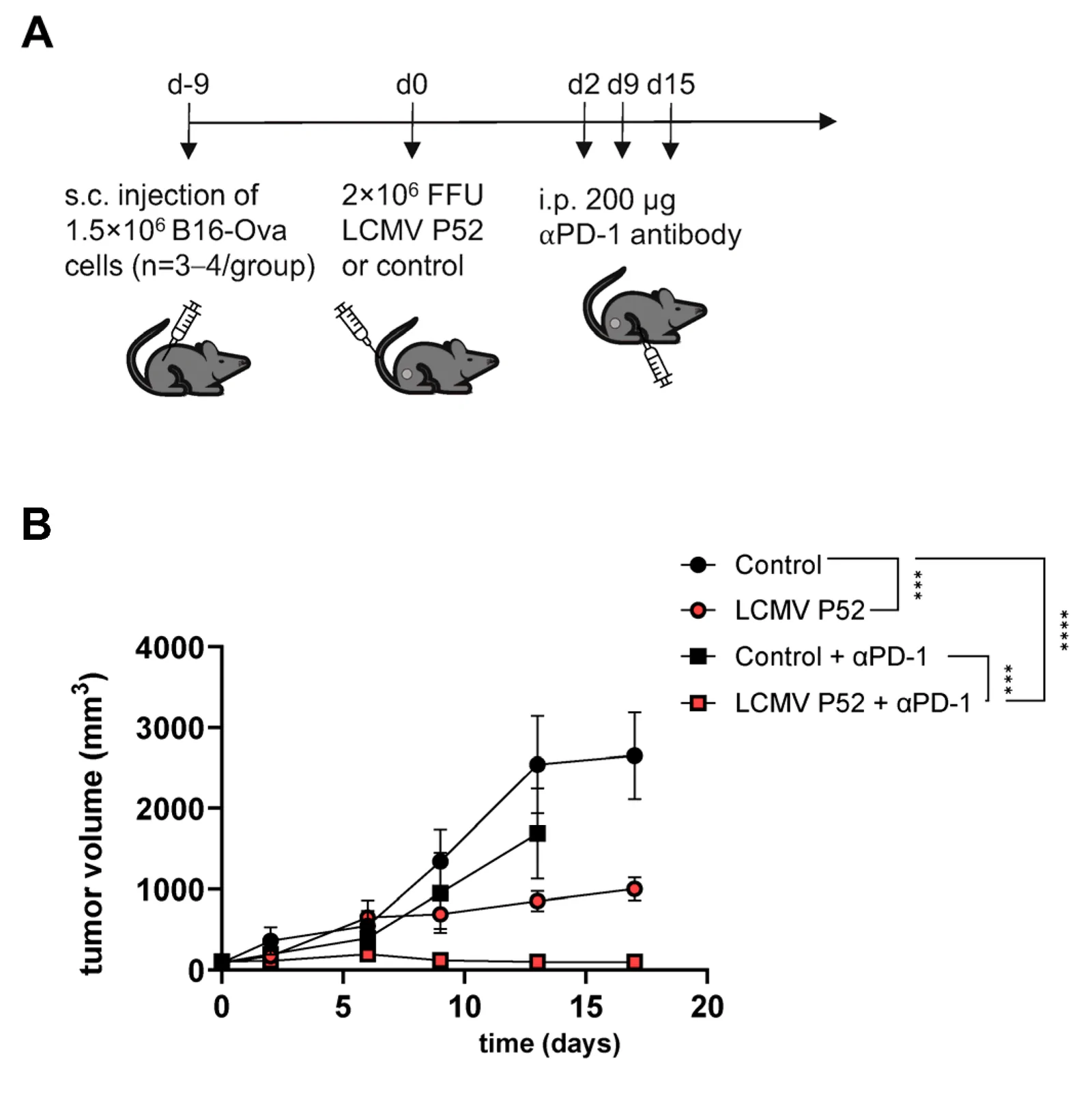
LCMV P52 sensitizes tumors to checkpoint blockade. (**A**,**B**) Female C57BL/6 mice were subcutaneously injected with 1.5 × 10^6^ B16-Ova tumor cells. On day 9 after tumor cell implantation, mice were left untreated (control, black symbols, *n* = 7) or intratumorally infected with 2 × 10^6^ FFU of LCMV P52 (red symbols, *n* = 7). Mice were separated into two groups and either left untreated (control: black circles, *n* = 3; LCMV P52: red circles, *n* = 3) or treated with 200 µg of anti-PD1 antibody i.p. on days 2, 9 and 15 (control: black squares, *n* = 4; LCMV P52: red squares, *n* = 4). Tumor volume (mm^3^) was measured until day 17 after infection. Mean ± SEM (**B**). Statistical analysis was performed with two-way ANOVA with Šidák correction for multiple comparisons and shows comparison on day 13 p.i. *** *p* ≤ 0.001, **** *p* ≤ 0.0001 values without statistical significance are not highlighted.

Our data suggest a marked involvement of adaptive immune effects in the LCMV P52 anti-tumoral activity. We hypothesized that the strong impact of LCMV P52 on tumor-infiltrating T cells may allow for the combination with checkpoint inhibition therapy. As LCMV P52 is effective against B16-Ova tumor metastasis formation (Figure 6), we employed the same subcutaneous tumor model to combine with anti-PD-1 treatment (Figure 7A). PD-1 checkpoint inhibition alone did not have a substantial effect on tumor growth (Figure 7B). Intra-tumoral LCMV P52 treatment resulted in a marked anti-tumoral effect (Figure 7B). In combination with checkpoint inhibition LCMV P52 virus administration showed more marked anti-tumor effects, leading to partial tumor regression (Figure 7B). In conclusion, treatment with LCMV P52 induces robust anti-tumor effects that are further enhanced in combination with checkpoint inhibition.

### 3.8. LCMV P52 Treatment Induces Only Transient Alterations in Serum Chemistry Parameters

We further sought to evaluate the safety profile of LCMV P52 therapy and determine whether treatment induces major adverse effects. Therefore, we analyzed a panel of serum chemistry parameters that are indicative of the functional status of different organs.

**Figure 8:**
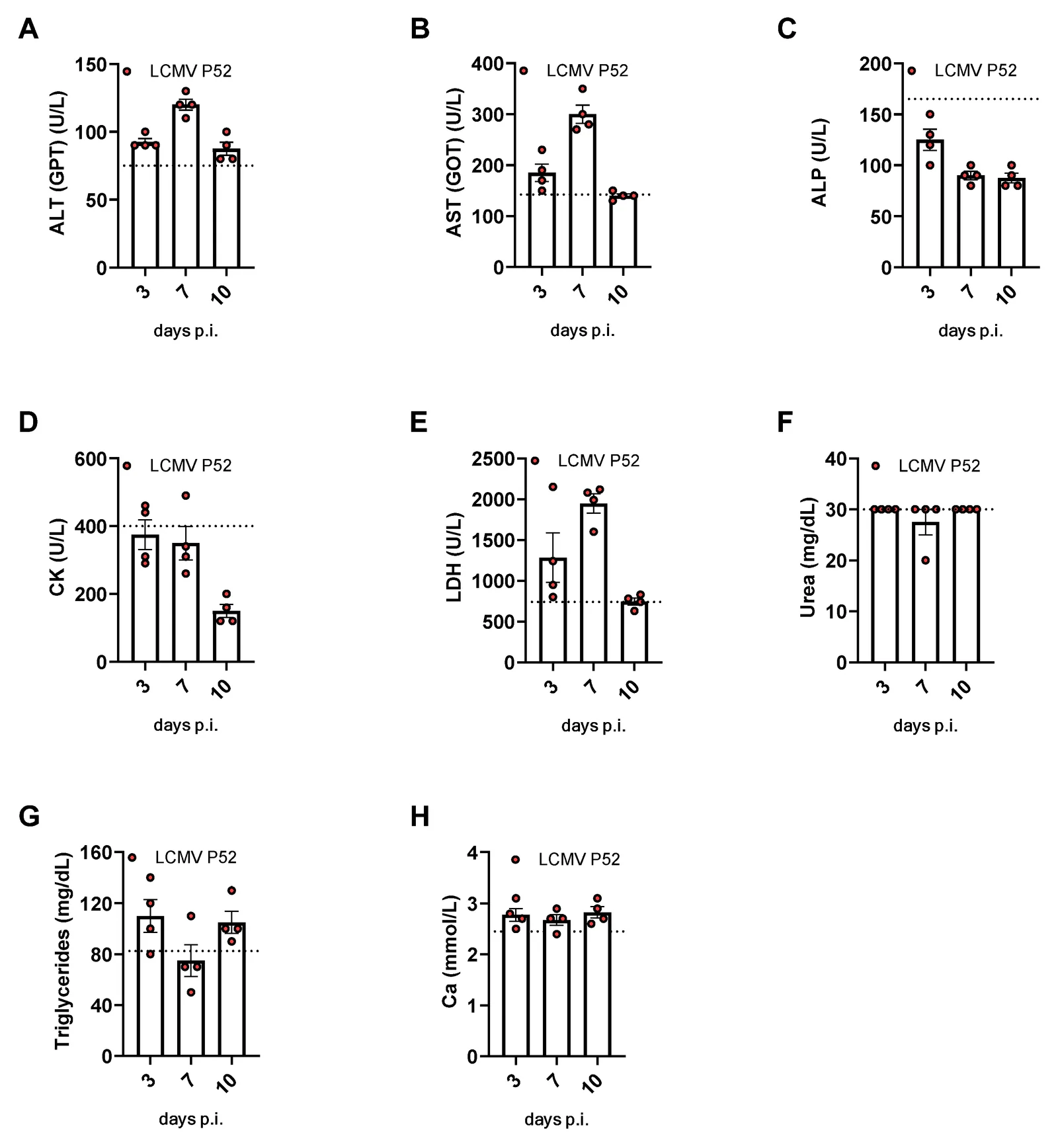
LCMV P52 treatment does not cause adverse effects in mice. (**A**–**H**) Female C57BL/6 mice were intravenously infected with 2 × 10^6^ FFU of LCMV P52 (*n* = 4). Displayed parameters were measured in serum on days three, seven and ten after infection. Dotted lines indicate upper normal levels of displayed parameters. Mean ± SEM. ALT: alanine aminotransferase, AST: aspartate aminotransferase, ALP: alkaline phosphatase, CK: creatine kinase, LDH: lactate dehydrogenase, and Ca: calcium.

To assess potential liver toxicity, a known concern associated with LCMV infection [37], serum alanine aminotransferase (ALT), aspartate aminotransferase (AST), and alkaline phosphatase (ALP) levels were measured (Figure 8A–C). Transient elevations in ALT and AST levels were observed at day 7 post-treatment, indicating a temporary hepatic insult. Importantly, both parameters returned to baseline levels by day 10 post-infection, suggesting complete resolution of liver injury. In contrast, the ALP levels remained unchanged throughout the study, providing no evidence of cholestatic liver damage. Potential muscle damage was evaluated by measuring serum creatine kinase (CK) levels (Figure 8D). The CK concentrations remained unchanged throughout the observation period, suggesting that LCMV P52 treatment did not induce muscle toxicity. In addition, the serum lactate dehydrogenase (LDH) levels were analyzed as a marker of general non-specific tissue damage (Figure 8E). A transient increase in LDH levels was observed following treatment, which subsequently normalized by day 10 post-infection. This finding is consistent with a temporary and reversible tissue response to viral infection. Renal function was assessed by measuring serum urea concentrations. No increase in urea levels was detected at any time point, indicating the absence of detectable kidney impairment associated with treatment (Figure 8F). Serum triglycerides were measured as an indicator of lipid metabolism (Figure 8G). Mild elevations were observed at day three post-infection; however, these changes were small in magnitude and likely reflect biological or experimental variability rather than a treatment-related adverse effect. Finally, the serum calcium levels remained stable throughout the study, providing no evidence for disturbances in mineral homeostasis (Figure 8H). Taken together, these data demonstrate that LCMV P52 treatment was generally well tolerated and induced only transient reversible alterations in selected serum chemistry parameters without evidence of sustained liver, kidney, muscle, or systemic toxicity.

## 4. Discussion

Our study demonstrates that the modified LCMV variant LCMV P52 elicits potent and multifaceted anti-tumor activities across divergent immunological landscapes, with the strongest efficacy observed in cold tumor models.

Across the models, LCMV P52 replicates preferentially within tumor tissues upon single systemic administration, providing a mechanistic basis for strong on-target inflammation with limited off-tumor exposure, consistent with other virus therapies showing that tumor-restricted viral tropism enhances local immune activation while minimizing systemic toxicity [38,39]. While most existing studies focus on oncolytic viruses, similar TME-restricted inflammatory conditioning has now been described for the non-lytic LCMV P52, which selectively engages tumor niches. In addition, LCMV elicits neutralizing antibody responses only with delayed kinetics, with detectable neutralizing activity often arising 30–60 days after infection. This characteristic, which has been linked to glycan-mediated shielding of neutralizing epitopes [40], may represent a particular advantage for cancer therapy as meaningful anti-tumoral activity can be achieved following a single intravenous administration that also enables access to distant metastatic lesions.

Following infiltration of the tumor, LCMV is detected by intracellular and endosomal PRRs, most notably RIG-I and TLR7, thereby activating anti-viral sensing pathways that drive potent innate immune induction within the tumor microenvironment [41,42]. The anti-viral immune response is characterized by a strong T cell component, which, in the context of the tumor, may contribute to sustained local viral persistence and antigen release. The innate inflammatory response promotes the recruitment of effector lymphocytes, including CD8^+^ T cells and NK cells, into the tumor microenvironment. Together with potentially enhanced cross-priming of tumor-specific T cells, this inflammatory milieu also supports the infiltration of tumor- and virus-specific T cells, further amplifying the local immune response.

Interestingly, it has been described that alteration of the LCMV glycoprotein can lead to increased receptor affinity and enhanced cross presentation due to higher stability of the GP trimer and retention in the ER [43]. Furthermore, replication capacity, tropism, and receptor affinity is affecting innate immune activation including IFN-I production and effectivity [27]. Although no published data currently link amino-acid positions 181 and 185 to increased GP stability, it is conceivable that such alterations could enhance GP robustness and thereby potentiate anti-tumoral T cell responses during LCMV P52 infection beyond affecting cellular virus infection [24]. Both amino acids are located within a variable surface-exposed region of GP1 that was previously identified as relevant for αDG receptor interaction, with an arginine at position 185 lowering the affinity to αDG compared to alanine [25]. But, in contrast to other mutations associated with high αDG affinity, the amino acid at position 185 had no impact when combined with the high-affinity mutant H155Y. This indicates that position 185 has a modulatory rather than essential role in αDG engagement. This is consistent with the observation that plasmacytoid dendritic cells (pDCs), although expressing high levels of αDG, are infected less effectively with LCMV P52 compared to wildtype LCMV WE [24]. Indeed, when analyzing the single mutations in recombinant viruses, we could show that mutations at positions 181 and 185 do increase viral entry but appear not to be substantially involved in αDG binding [16]. These two mutations rather make virus infection more dependent on the virus entry co-receptor CD164 [44], providing an explanation for the modulatory role in infection of these mutations via CD164. The observed accumulation of highly functional CD8^+^ effector T cells accompanied by increased dendritic cell presence further suggests functional reinvigoration, consistent with reports that inflammatory viral conditioning promotes effector differentiation and mitigates T cell dysfunction within suppressive TMEs. Importantly, enhancing effector T cell functionality together with dendritic cell activation has been shown to reshape the TME toward a more immunostimulatory and less immunosuppressive state, strengthening cytotoxic responses and reducing regulatory and myeloid-derived suppressive programs, thereby supporting more effective anti-tumor immunity [45]. In line with this, our observation of reduced myeloid-cell infiltration and fewer regulatory T cells suggests a shift away from immunosuppressive myeloid and Treg-dominated niches. Such remodeling of the cellular composition of the TME is likely to further lower inhibitory pressure, enhance antigen presentation, and facilitate sustained CD8^+^ T cell activity, thereby promoting a more permissive environment for effective anti-tumor immunity [46,47].

The observed immunological changes have direct implications for rational combination therapies. By increasing both T cell numbers and their effector quality, LCMV P52 may convert immunological cold TMEs into more permissive TMEs to enhance immune checkpoint blockade, aligning with evidence that inflammation improves ICI (immune checkpoint inhibitor) responsiveness by expanding functional tumor-reactive CD8^+^ T cell subsets [48]. Similarly, the observed synergy with adoptive T cell therapy is supported by reports that virally inflamed TMEs promote engraftment, expansion, and cytotoxic function of transferred T cells [49]. Thus, LCMV P52 appears to act as a potent microenvironmental adjuvant, bridging innate sensing, antigen presentation, and effector T cell recruitment, a conceptual framework proposed for next-generation virotherapies.

Importantly, while cold tumors showed the most pronounced benefit of LCMV P52 virotherapy, hot tumors such as MC38-Ova and CT-26 also responded. This pattern aligns with observations that viral immune modulation can additively enhance pre-existing immunity rather than requiring an immunologically naïve TME [50]. These findings position LCMV P52 as a versatile virotherapy platform that is capable of boosting anti-tumor immunity across diverse tumor immune phenotypes. Importantly, the therapeutic potential of LCMV P52 is complemented by a favorable safety profile, and treatment was associated with only minimal adverse effects, which were primarily limited to the liver. These observations are consistent with previous efforts to improve the therapeutic window of arenavirus-based virotherapy through rational virus engineering. Notably, LCMV P52 represents an intermediate stage in the development of the recombinant virus WE-CL13-GP181M-185W-492I, which combines tumor-tropic mutations identified during serial passaging with the attenuating WE-CL13 backbone, which was recently described [16]. This optimized recombinant virus exhibited enhanced propagation in tumor cells while maintaining limited replication in healthy tissues and a favorable safety profile in preclinical models [16]. Furthermore, the combination of mutations from different LCMV strains resulted in increased attenuation, with viral replication in the liver being almost completely abolished and replication in the lung remaining low. Together, these findings suggest that tumor tropism and safety can be further improved simultaneously through the combination of attenuating and tumor-targeting mutations. Looking forward, additional strategies could further increase the safety of arenavirus-based therapies. One promising approach would be the incorporation of tissue-specific microRNA target sequences into the viral genome to selectively suppress viral replication in sensitive organs while preserving replication within tumors, thereby further widening the therapeutic window.

## Data Availability

The data presented in this study are available on request from the principal investigator (PI), Jörg Vollmer.

## Supplementary Materials

The following supporting information can be downloaded at: https://www.mdpi.com/article/10.3390/v18090998/s1, Figure S1: LCMV P52 reprograms the immunogenic CT-26 tumor microenvironment.
