# Supplementary material for: Lymphocytic Choriomeningitis Virus Bearing GPC Mutations for Enhanced Tumor Tropism and Strong Anti-Tumor Activity
Source: Viruses. 2026 Sep 11;18(9):998. doi: 10.3390/v18090998 (PMC13611857; doi:10.3390/v18090998)
Supplement: Supplementary file 1 [file viruses-18-00998-s001.zip › viruses-4404489-supplementary.pdf]

Supplementary Figure S1

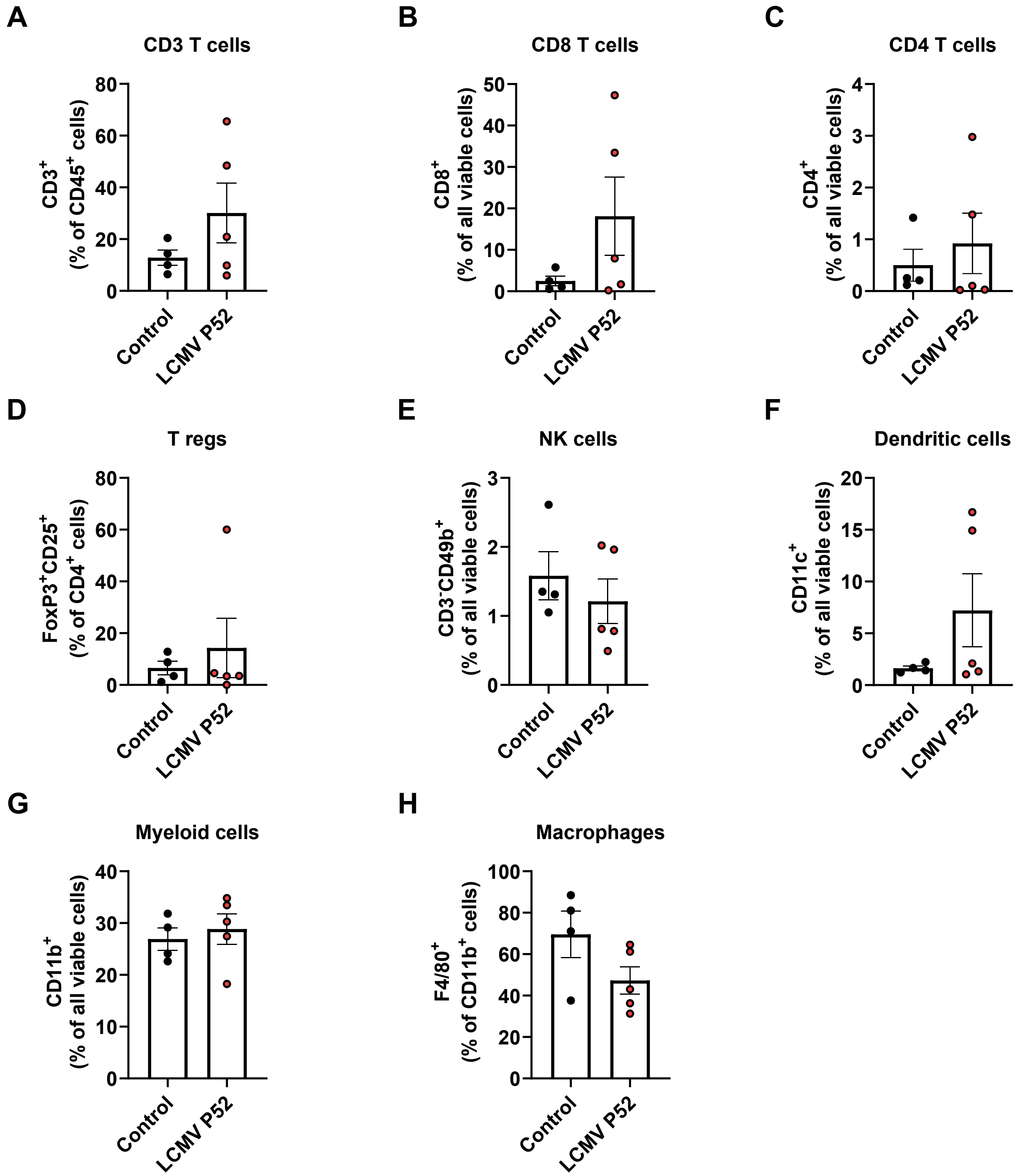

Supplementary Figure S1. LCMV P52 reprograms the immunogenic CT26 tumor microenvironment **(A–H)** Female BALB/c mice (*n* = 5) were injected subcutaneously with 1×10^6 CT26 cells and treated intravenously with PBS or 2×10^4 FFU of LCMV P52. 11 days after treatment mice were sacrificed and tumor tissue was analyzed by flow cytometry to determine the percentages of $CD3^+$, $CD4^+$, $CD8^+$, Treg, NK cell, dendritic cell, myeloid, and macrophage populations. Unpaired two-tailed Welch's t test **(A–H)**. Non-significant differences are not indicated.
